# Effects of Coronavirus Disease 2019 (COVID-19) on Peripheral Blood Lymphocytes and Their Subsets in Children: Imbalanced CD4^+^/CD8^+^ T Cell Ratio and Disease Severity

**DOI:** 10.3389/fped.2021.643299

**Published:** 2021-04-14

**Authors:** Shima Mahmoudi, Bahareh Yaghmaei, Meisam Sharifzadeh Ekbatani, Babak Pourakbari, Amene Navaeian, Nima Parvaneh, Mohammad Taghi Haghi Ashtiani, Setareh Mamishi

**Affiliations:** ^1^Pediatric Infectious Disease Research Center, Tehran University of Medical Science, Tehran, Iran; ^2^Division of Pediatric Intensive Care Unit, Pediatrics Center of Excellence, Children's Medical Center, Tehran University of Medical Sciences, Tehran, Iran; ^3^Department of Infectious Diseases, Pediatrics Center of Excellence, Children's Medical Center, Tehran University of Medical Sciences, Tehran, Iran; ^4^Research Center for Immunodeficiencies, Pediatrics Center of Excellence, Children's Medical Center, Tehran University of Medical Sciences, Tehran, Iran; ^5^Department of Pathology, Pediatrics Center of Excellence, Children's Medical Center, Tehran University of Medical Sciences, Tehran, Iran

**Keywords:** lymphocyte, children, COVID-19, SARS-CoV-2, CD4+/CD8+ lymphocytes

## Abstract

**Introduction:** While pathogenesis in COVID-19 is not fully known and the effects between SARS-CoV-2 and the immune system are complicated, it is known that lymphopenia, hyper-inflammatory responses, and cytokines play an important role in the pathology of COVID-19. While some hematological abnormalities have been described among the laboratory features of COVID-19, there have not been studies reported on lymphocyte subset analyses in children. The aim of this study was to describe lymphocyte subsets in pediatric patients with mild/moderate or severe COVID-19.

**Methods:** The subjects in the study were children with severe acute respiratory syndrome coronavirus 2 (SARS-CoV-2) pneumonia confirmed with the real-time RT-PCR. The subjects were admitted to the Children's Medical Center, affiliated with the Tehran University of Medical Sciences, between March 7^th^ and June 10^th^ of 2020. The complete blood counts and lymphocyte subpopulations were analyzed for each patient.

**Results:** The study included 55 hospitalized patients with confirmed SARS-CoV-2 infection (34 patients (62%) with an observed mild/moderate case of the disease and 21 patients (38%) with severedisease). Lymphocyte counts were found to be lower in patients with a severe case (mean ± SD 1.6 ± 0.9 in the severe group vs. 2.3 ± 2.2 in the mild group). Compared to the group with mild/moderate pneumonia, children with severe pneumonia had an increased count of CD8^+^ T cell and a lower percentage of CD4^+^ T cell. However, the differences between the groups were negligible. Interestingly, the severe group had a lower CD4^+^/CD8^+^ T cell ratio compared to the mild group (1.1 ± 0.47 vs. 1.4 ± 0.8, *p*-value: 0.063). CD4^+^/CD8^+^ T cell ratio <2, 1.5, and 1 was found in 48 (87%), 40 (73%), and 19 cases (35%). All of the seven cases in which the subject passed (13%) had CD4^+^/CD8^+^ T cell ratio of <2, 86% had CD4^+^/CD8^+^ T cell ratio of <1.5, and 29% had CD4^+^/CD8^+^ T cell ratio of <1.

**Conclusion:** The CD4^+^/CD8^+^ T cell ratio was lower in patients with severe COVID-19 compared to those with mild/moderate form of disease. However, although a decline in CD4^+^/CD8^+^ ratio may serve as a useful metric in analyzing of the derangement in immune responses in patients with severe COVID-19, further study with larger sample sizes is highly recommended.

## Introduction

The Coronavirus Disease 2019 (COVID-19), which is caused by severe acute respiratory syndrome corona virus 2 (SARS-CoV-2), has caused an on-going global pandemic that rapidly spread from China (1). All people, including children, are susceptible to SARS-CoV-2, and a rapid increase in both morbidity and mortality rate have been observed due to human-human transmission of the virus (2).

While pathogenesis in COVID-19 is not fully known and the effects between SARS-CoV-2 and the immune system are complicated, it is known that lymphopenia, hyper-inflammatory responses, and cytokines play an important role in the pathology of COVID-19 (3–5).

Recent studies suggest that T lymphocytes, particularly CD4^+^ T and CD8^+^ T cells, are affected by SARS-CoV-2, CD4^+^ and CD8^+^ T cells play a fundamental role in controlling viral infections and maintaining cellular, humoral and cytotoxic immune responses. They may play a very important role the pathological process of COVID-19 (6, 7). CD4^+^ T cells have numerous roles and are required to support CD8^+^ T cell responses. Moreover, these cells help B cells to elicit antibody responses (8). CD8^+^ T play critical roles in mediation of viral clearance and acute viral respiratory infections in viruses such as respiratory syncytial virus, influenza A virus, and human metapneumovirus (9).

A number of hematological abnormalities have been observed in the laboratory features of COVID-19 (7, 10–13). Several studies concerning changes of lymphocyte subsets and their correlation with the severity and outcome of the disease have been reported in adults (10, 14, 15). However, there are no studies on lymphocyte subset analyses in pediatric patients. This study seeks to describe the characteristics of lymphocyte subsets in pediatric patients with both mild/moderate and severe COVID-19. This study aimed to evaluate the lymphocyte subsets in 55 laboratory-confirmed cases with COVID-19.

## Materials and Methods

This study received ethical approval (IR.TUMS.VCR.REC.1399.060) from the Tehran University of Medical Sciences in Tehran, Iran. All participants gave written informed consent, and the study was carried out following the guidelines of the Declaration of Helsinki.

### Patient Selection

The subjects of the study were hospitalized children with SARS-CoV-2 pneumonia confirmed using SARS-CoV-2 real-time RT-PCR of nasopharyngeal swab samples according to the CDC diagnostic panel (12, 16). The subjects were admitted to the Children's Medical Center, an Iranian referral hospital, between March 7th and June 10th of 2020. Each subject underwent the process of detection of peripheral blood lymphocyte subsets. Information recorded for each patient includes: age, sex, underlying disease, intensive care unit (ICU) admission, the need for invasive mechanical ventilation, laboratory findings, chest computed tomography (CT), and mortality. All laboratory tests were interpreted based on reference normal range of Children's Medical Center. Chest CT scans were reported by an expert radiologist, and an abnormal CT finding was considered if typical CT imaging featured: peripheral, bilateral, or ground-glass opacification with or without consolidation or visible intralobular lines, multifocal ground-glass opacification or rounded morphology with or without consolidation or visible intralobular lines, reverse halo signs or any other findings of organizing pneumonia were reported (17). The severity of COVID-19 was categorized to two groups (severe/critical vs. mild/moderate) according to the clinical findings, severity of pneumonia, respiratory failure, shock, and other organ failures (18). A severe/critical type was defined on admission according to the following criteria: (1) a breathing rate of ≥30 times/min; (2) pulse oximeter oxygen saturation (SpO_2_) ≤93% at rest; and (3) ratio of the partial pressure of arterial oxygen (PaO_2_) to the fraction of inspired oxygen (FiO_2_) ≤300 mmHg (9).

### Real-Time RT-PCR for SARS-CoV-2

Nasopharyngeal samples were taken from the patients and tested for SARS-CoV-2; however, in infants that collection of nasopharyngeal samples were so difficult, throat swab specimens were obtained from the upper respiratory tract. The rRT-PCR assay was performed in accordance with the CDC protocol using the same primers and probes of N1 and N2 and RNase P (RP) as an internal control (10). The rRT-PCR assay was performed following recommended cycling conditions: reverse transcription at 55°C for 5 min and 95°C for 5 min, followed by 45 cycles of PCR at 95°C for 15 s and 58°C for 15 s. The cycle threshold (Ct) value above 37.0 was considered negative.

### Flow Cytometry for Determination of Lymphocyte Subsets

Flow cytometry analysis was used for the detection of lymphocyte subsets. Samples of EDTA anticoagulated peripheral blood (2 ml) was obtained from patients with COVID-19 before initiation of treatment. All samples were tested within 6 h of being obtained, and CD4^+^ and CD8^+^ T-cell counts (cells/μl) were measured using multiple-color flow cytometry. The cells were analyzed on a BD FACS Canto II flow cytometry system (BD Biosciences). Lymphocyte subset percentage was categorized in three groups including below normal range, within normal range and above normal range according to the age of the patients (19).

### Statistical Analysis

Statistical analysis was performed with the Statistical Package for the Social Sciences (SPSS version 18.0, SPSS Inc., Chicago, IL, USA).

Categorical data was described using percentages and continuous data as median with interquartile range (IQR). Normally distributed continuous variables were presented as means with standard deviations (SD). A comparison test of the differences between the two groups was conducted using the *t*-test, Chi-square test, or Mann–Whitney *U* test. Logistic regression analyses were performed to evaluate factors associated with in-hospital mortality and severity of the disease. Variables with a two-tailed *p*-value < 0.05 were considered statistically significant.

## Results

### Baseline Data

The study population consisted of 55 hospitalized patients with confirmed SARS-CoV-2 infection. The age of the patients ranged from 20 days to 14.0 years (6.9 ± 4.1). Thirty-four patients (17 males and 17 females) with a mean age of 7.0 ± 3.9 years had mild/moderate infection, and 21 patients (17 males and 4 females) with a mean age of 7.0 ± 4.6 years were included in the severe group (Table 1). The severe form of the disease was significantly more common in males than females (81 vs. 19%, *p*-value = 0.022).

**Table 1:** Demographic, clinical, laboratory and radiological findings of patients with mild/moderate and severe COVID-19.

**Variables**		**Mild/moderate**	**Severe**
		**(*n* = 34)**	**(*n* = 21)**
Age (year)		7.0 ± 3.9	7.0 ± 4.6
Sex (male)		17 (50)	17 (81)
Underlying disease		10 (29)	14 (67)
ICU admission		2 (6)	7 (33)
Requiring a ventilator		0	3 (14)
Abnormal CT findings		14 (67)	16 (89)
Death		3 (9)	4 (19)
**Clinical findings**			
Fever		26 (76)	16 (76)
Cough		20 (59)	15 (71)
Vomiting		15 (44)	3 (14)
Abdominal pain		9 (26)	4 (19)
Diarrhea		10 (29)	2 (10)
Myalgia		12 (35)	5 (24)
Tachypnea		15 (44)	15 (71)
**Laboratory findings**			
Elevated ESR		25 (81)	17 (85)
Elevated C-reactive protein		24 (71)	17 (81)
White blood cell count	<4 × 10^9^/L	4 (12)	3 (14)
	4–10 × 10^9^/L	24 (70.5)	15 (72)
	>10 × 10^9^/L	7 (20.5)	5 (24)
Neutrophil count	<2 × 10^9^/L	3 (9)	2 (9)
	2–7 × 10^9^/L	24 (70.5)	14 (67)
	>7 × 10^9^/L	7 (20.5)	5 (24)
Lymphocyte count	<0.8 × 10^9^/L	13 (38)	6 (29)
	0.8–4 × 10^9^/L	20 (59)	15 (71)
	>4 × 10^9^/L	1 (3)	0
Platelet count	<150 × 10^9^/L	8 (23)	6 (29)
	150–450 × 10^9^/L	2 (59)	13 (62)
	>450 × 10^9^/L	6 (18)	2 (9)
CD4+ T cells	Below the normal values	10 (29)	8 (38)
	Within the normal value	21 (62)	13 (620
	Above the normal values	3 (9)	0
CD8+ T cells	Below the normal values	4 (12)	1 (5)
	Within the normal value	24 (70.5)	14 (67)
	Above the normal values	6 (17.5)	6 (28)

Among all patients, nine cases were admitted to the ICU, and seven of them had severe pneumonia (33% in severe group and 6% in mild/moderate group, *p*-value = 0.02). One of these two patients with mild/moderate disease had rhabdomyosarcoma of the nasopharynx and the other was referred to ICU following suspicion of peritonitis. Three patients received invasive mechanical ventilation. The presence of underlying diseases was significantly higher in patients with severe cases compared to that of patients with mild/moderate disease [67% (*n* = 14) vs. 29% (*n* = 10), *p*-value = 0.007]. The underlying diseases of patients with severe disease were cancer (*n* = 5), cardiovascular diseases (*n* = 5), neurodevelopmental disorders (*n* = 1), cystic fibrosis (*n* = 1), chronic hypertension (*n* = 1) and immunodeficiency diseases (*n* = 1). In patients with mild/moderate disease, the underlying comorbidities included cancer (*n* = 4), cardiovascular diseases (*n* = 2), rhabdomyosarcoma of the nasopharynx (*n* = 1), lopus (*n* = 1), Down syndrome (*n* = 1), and hydrocephaly (*n* = 1). Abnormal CT findings were found in 89 and 67% of cases with severe and mild/moderate forms of the disease, respectively (*p*-value = 0.139). The mortality was 13% (*n* = 7). Only one deceased case had no comorbidities. The underlying diseases of deceased patients were cancer, cardiovascular diseases, chronic hypertension, cystic fibrosis, immunodeficiency diseases, and Down syndrome.

### Laboratory Findings

Inflammatory indicators, C-reactive protein (CRP), and erythrocyte sedimentation rate (ESR) were abnormal in 17 (85%), and 17 (81%) patients in severe, and 25 (81%), and 24 (71%) patients in mild/moderate group (Table 1).

In blood tests of cases with severe pneumonia, leukocytes, lymphocytes, and neutrophils were below the normal range in 3 (14%), 6 (29%), and 2 (9%) patients and above the normal range in 3 (14%), 0 (0%), and 5 (24%) patients, respectively. In another group with mild/moderate disease, elevated leukocytes, lymphocytes, and neutrophils counts were seen in 6 (17.5%), 1 (3%), and 7 (20.5%) patients, while it was below the normal range in 4 (12%), 13 (38%), and 3 (9%) patients, respectively. There were no significant differences in white blood cell (WBC) counts, Red Blood Cells (RBC), hemoglobin, platelet, and neutrophil counts between the two groups. Lymphocyte counts were lower in patients with severe forms of the disease (mean ± sd 1.6 ± 0.9 in the severe group vs. 2.3 ± 2.3 in the mild/moderate group, *p*-value = 0.052) (Table 2).

**Table 2:** The patients' laboratory findings and blood lymphocyte subsets of children with COVID-19.

**Parameter**	**Mild/moderate**	**Severe**	***P*-value**
	**(mean ± SD)**	**(mean ± SD)**	
White blood cell count	8.8 ± 6.1	8.6 ± 4.3	0.4
(× 10^9^ cells per L)			
Neutrophil count (× 10^9^ cells per L)	5.8 ± 5.0	6.2 ± 3.9	0.69
Lymphocyte count (× 10^9^ cells per L)	2.3 ± 2.2	1.6 ± 0.9	0.052
Platelet count (× 10^9^ cells per L)	272.5 ± 159	270 ± 156	0.9
Red Blood Cells (× 10^9^ cells per L)	4.4 ± 0.7	4.4 ± 0.1	0.19
Hemoglobin (g.dL)	11.9 ± 1.9	11.1 ± 2.6	0.32
CD4^+^ Tcells %	34.8± 11.2	32 ± 8.6	0.34
CD8^+^ T cells %	27.7 ± 10.1	31.3 ± 8.1	0.18
CD4^+^/ CD8^+^ T cells ratio	1.46 ± 0.8	1.1 ± 0.47	0.063
C-reactive protein (mg.L)^*^	14 (4-62)	34 (7.2-69)	0.22
Erythrocyte sedimentation rate (mm/h)^*^	22 (14-41)	40.5 (23.5-58)	0.03

* *Median (Interquartile range)*.

### Lymphocyte Subsets in Peripheral Blood of Patients

The mean time from the onset of symptoms to the testing of lymphocyte subsets was 4.8 ± 3.8 days. According to the results of each index, CD4^+^ T and CD8^+^ T cells were divided into three groups: below the normal values, within the normal value, and above the normal values. The corresponding values and proportions were respectively calculated based on age. The results are shown in Table 1.

Compared to the mild/moderate cases, patients with severe pneumonia had an increased count of CD8^+^ lymphocyte with a decreased CD4^+^ lymphocyte count. However, there were no significant differences in the percentage of CD4^+^ and CD8^+^ lymphocytes between the two groups (Table 2). No significant differences were found between the special features of CD4^+^ and CD8^+^ lymphocyte in different age among different group (*p*-value = 0.26 and *p*-value = 0.8 in mild/moderate group and *p*-value = 0.52 and *p*-value = 0.43 in severe group, respectively).

CD4^+^/CD8^+^ T cell ratio and COVID-19 Severity

Interestingly, patients with severe cases had a lower CD4^+^/CD8^+^ T cell ratio compared to patients with mild/moderate cases (1.1 ± 0.47 vs. 1.4 ± 0.8, *p*-value: 0.063) (Table 2).

A CD4^+^/CD8^+^ value of <2, 1.5, and 1 was found in 48 (87%), 40 (73%), and 19 cases (35%), respectively. The frequency of an inverted CD4^+^/CD8^+^ T cell ratio increased with age. A ratio of <1 was seen in 32% of patients less than 5 years (*n* = 6) and in 68% of 5 to 15-year-olds (*n* = 13). However, the mean value of CD4^+^/CD8^+^ T cell ratio was not significantly different in cases with different age group (*p*-value: 0.15). Males were more likely to have a decreased ratio than females (Table 3).

**Table 3:** Demographic and laboratory findings of children with COVID-19 according to different CD4^+^/CD8^+^ T cells ratio.

**Parameter**		**CD4**^****+****^**/CD8**^****+****^ **T cells ratio**							
		**<** **1 (*****N*** **=** **19)**		** <1.5 (*****N*** **=** **40)**		** <2 (*****N*** **=** **48)**		**≥2 (*****N*** **=** **7)**	
		***N***	**%**	***N***	**%**	***N***	**%**	***N***	**%**
Mild		9	47	23	57.5	28	58	6	86
Severe		10	53	17	42.5	20	42	1	14
Sex	Male	12	63	26	65	31	65	3	43
	Female	7	37	14	35	17	35	4	57
Age	<1 year	1	5	2	5	2	4	2	29
	1 to 5 year	5	26	12	30	17	35	3	43
	5 to 10 year	6	32	16	40	17	35	2	29
	10 to 15 year	7	37	10	25	12	25	0	0
Death		2	11	6	15	7	15	0	0
Underlying disease		10	53	20	50	23	48	1	14
Abnormal CT findings		10	53	21	52.5	26	54	4	57
ICU admission		4	21	8	20	8	17	1	14
Invasive mechanical ventilation		2	11	3	7.5	3	6	0	0
White blood cell count	<4 × 10^9^/L	3	16	6	15	7	15	0	0
	4–10 × 10^9^/L	13	53	27	67.5	34	71	5	71
	>10 × 10^9^/L	3	16	7	17.5	7	15	2	29
Neutrophil count	<2 × 10^9^/L	3	16	5	12.5	5	10	0	0
	2–7 × 10^9^/L	11	58	25	32.5	33	69	5	71
	>7 × 10^9^/L	5	26	10	25	10	21	2	29
Lymphocyte count	<0.8 × 10^9^/L	6	32	14	35	16	33	3	43
	0.8–4 × 10^9^/L	13	53	25	32.5	31	62.5	4	57
	>4 × 10^9^/L	0	0	1	2.5	1	2	0	0
Platelet count	<150 × 10^9^/L	5	26	11	27.5	13	27	1	14
	150–450 × 10^9^/L	12	63	23	57.5	28	58	5	71
	>450 × 10^9^/L	2	11	6	15	7	15	1	14
C-reactive protein	>6 mg/L	15	79	30	75	36	75	5	71
Erythrocyte sedimentation rate	>10 mm/h	14	74	30	75	37	77	5	71

All of the seven cases in which the subject passed away (13%) had CD4^+^/CD8^+^ T cell ratio of <2, 86% had CD4^+^/CD8^+^ T cell ratio of <1.5, and 29% had CD4^+^/CD8^+^ T cell ratio of <1. According to the logistic regression analysis, underlying diseases were identified to be independent risk factors associated with the severity of disease (OR = 3.6; *p*-value = 0.042), after adjusting for confounding factors including age, sex, and CD4^+^/CD8^+^ T cell ratio. Moreover, by logistic regression analysis, no significant association between underlying diseases and in-hospital mortality was found, after adjusting for different factors including age, sex, underlying diseases, and CD4^+^/CD8^+^ T cell ratio (*p*-value >0.05).

## Discussion

To our knowledge, this is the first preliminary study that evaluates the lymphocyte subset characteristics in children with COVID-19.

Although earlier studies reported that children were less likely to become infected with COVID-19 than other age groups, recent studies revealed that children are at a similar risk of infection to the general population (2, 20). Consistent with previous reports (5, 21–23), this study exhibits a substantial a male predominance in COVID-19 infection.

Changes in the immune system may result in a decrease in immune function as well as increased severity, morbidity, and mortality of infections (1, 2). Similar to the previous reports in children, the laboratory findings showed that normal leukocytes counts were more common in cases with severe and mild forms of the disease (24). It has been hypothesized that the repletion of lymphocytes plays a vital role in the recovery of patients with COVID-19 (25). Lymphopenia and cytokine storm in exceedingly pathogenic coronavirus infections, such as SARS coronavirus (SARS-CoV), MERS coronavirus (MERS-CoV), and SARS COV-2 infections might be associated with disease severities (26).

Although on average 63–84.6% of adult patients with severe disease forms have lymphopenia (27, 28), it was found in 19 children in our study (34.5%). In severe cases of the diseases, lymphocyte counts were significantly lower in patients with severe forms of the disease. A significantly low value of lymphocytes may denote clinical worsening and increased risk of a poor outcome in some cases. According to previous reports in children with probable or suspected SARS (29, 30), total lymphopenia was common and found to be increasingly prominent in older children with more severe forms of the disease. The counts of total leukocytes, RBCs, platelets, and neutrophils were not significantly different in severe and mild/moderate groups.

Inflammatory markers, including CRP and ESR, were higher in patients with severe cases compared to those in mild/moderate cases, demonstrating a higher inflammatory state during severe infection. Since cytokine storm is associated with apoptosis of lymphocytes, a decrease in the number of peripheral lymphocytes in severe cases is probable (31).

Although an apparent decline in peripheral lymphocytes in COVID-19 patients has been reported, alteration in the T cells subsets is still unclear (9). However, it has been reported that CD4^+^ T cells and CD8^+^ T cells decreased more in the severe cases of COVID-19 than in the mild cases (9, 27, 31). In our study, higher numbers of CD8^+^ T cells were documented in the severe group compared to the mild/moderate group. The CD4^+^ lymphocytopenia was more prevalent in the severe cases than in the mild cases (38 vs. 29%). On the other hand, a higher percentage of CD8^+^ T cells was found in the severe cases (28%) than in the mild cases (17.5%). However, these differences are not significant. Among 25 mild/moderate COVID-19 cases in the study of Bai et al. (24), 23 had normal CD8^+^ T lymphocyte counts, while increased CD8^+^ T lymphocyte counts were mildly increased in two cases (8%).

Increasing CD4^+^ T cell loss and an increase in CD8^+^ T cells are consistent features of HIV-1 infection. It has been reported that CD8^+^ T cells' count may change slightly, while the CD4^+^ T cells count decline at an inconstant rate. A progressive decline in CD4^+^ T cells, and a rise in CD8^+^ T cells, suggests a rise in CD8^+^ T cells to compensate for the loss of CD4^+^ T cells (32).

Although lymphocyte count, especially CD4^+^, has been reported as a clinical indicator of disease severity (7), no significant differences between CD4^+^ T cells and CD8^+^ T cells and severity of disease were found in our study. Alternatively, the CD4^+^/CD8^+^ T cell ratio was lower in patients with severe disease compared to those with mild/moderate cases of disease.

Li et al. reported that in adults with more serious disease, CD4^+^ T cell and CD8^+^ T cell counts are closely related to the severity of disease (15). In contrast to previous reports in adults where the majority of severe COVID-19 cases (6, 33) were recorded with absolute numbers of total T lymphocytes, CD4^+^ T cells, and CD8^+^ T cells below the normal limit, we demonstrated that the percentage of CD4^+^ T cells, and CD8^+^ T cells were reduced below the normal limit in 29 and 38% of cases with mild/moderate and severe COVID-19 cases, respectively. However, CD8^+^ T cells were reduced below the normal limit in 12 and 5% of cases with mild/moderate and severe COVID-19, respectively. According to the recent meta-analysis, no significant association of the in the subsets of CD4+ T cells and CD8+ T cells with COVID-19 progression and mortality was found (34).

The low frequency of lymphopenia in severe cases is mainly linked to the substantial decrease in absolute T cell counts, especially CD8^+^ T cells (27). Therefore, low rate of lymphopenia in children might be due to the fact that a majority of children have normal or even elevated CD8^+^ T cell counts.

The normal CD4^+^/CD8^+^ ratio in healthy patients is not well-defined. Ratios between 1.5 and 2.5 are typically regarded as normal (35). Low CD4^+^/CD8^+^ T cell ratios are mainly observed in HIV. However, a lower or inverted CD4^+^/CD8^+^ T cell ratio can also be linked with systemic lupus erythematosus, chronic inflammation, and cytomegalovirus infection as well (35, 36). CD8^+^ T cells are an important protective component against viral infections. Cytomegalovirus has a significant influence on the CD4^+^/CD8^+^ T cell ratio by increasing circulating CD8^+^ T cells in response to the infection (37).

Inversion of the CD4^+^/CD8^+^ T cell ratios is associated with low survival rates (38). A link between a low ratio and poor outcomes was observed in our study, and importantly, an 86% of death (*n* = 6) was reported in cases with low CD4^+^/CD8^+^ T cell ratios (<1.5). Calvet et al. showed a 45% decrease in median CD4^+^/CD8^+^ T cell ratio [1.7, (1.7 IQR) vs. 3.1 (2.4 IQR)] in critical patients compared to non-critical COVID-19 adult patients (39).

According to the previous reports, most cases without underlying diseases experience a mild disease and children with comorbidities might show severe symptoms (40, 41). We found 3.6-fold increased risk of the severity of disease in patients with underlying diseases, while no significant association between underlying diseases and in-hospital mortality was found, after adjusting for different factors including age, sex, underlying diseases, and CD4^+^/CD8^+^ T cell ratio.

In conclusion, the CD4^+^/CD8^+^ T cell ratio was lower in patients with severe COVID-19 compared to those with mild/moderate form of disease. Therefore, a decline in CD4^+^/CD8^+^ T cell ratio may serve as a useful metric for reflecting the derangement of immune responses and even mortality in patients with severe COVID-19. However, we should remind the facts that there are many unsolved issues on COVID-19, including the role of T cells in early lung lesions with lymphopenia, the causes of different clinical phenotype among individuals, immunopathogenesis of lung injury in SARS-CoV-2 infection (42); therefore, further study with larger sample sizes is highly recommended.

## Data Availability Statement

The raw data supporting the conclusions of this article will be made available by the authors, without undue reservation.

## Ethics Statement

The studies involving human participants were reviewed and approved by Tehran University of Medical Sciences. Written informed consent to participate in this study was provided by the participants' legal guardian/next of kin.

## Author Contributions

All authors listed have made a substantial, direct and intellectual contribution to the work, and approved it for publication.

## Conflict of Interest

The authors declare that the research was conducted in the absence of any commercial or financial relationships that could be construed as a potential conflict of interest.
